# Evaluating the effect of a smartphone-based self-help intervention on the quality of life of patients with acute coronary syndrome

**DOI:** 10.1186/s12872-025-05060-5

**Published:** 2025-08-20

**Authors:** Faezeh Setoudeh, Zahra Molazem, Giti Setoodeh, Parvin Ghaemmaghami

**Affiliations:** 1 Department of Medical-Surgical Nursing, School of Nursing and Midwifery, Shiraz University of Medical Sciences, Shiraz, Iran; 2 Psychiatric and Mental Health Nursing Department, School of Nursing and Midwifery, Shiraz University of Medical Sciences, Shiraz, Iran; 3 Department of Biostatistics, School of Nursing and Midwifery, Shiraz University of Medical Sciences, Shiraz, Iran

**Keywords:** Self-help intervention, Quality of life, Acute coronary syndrome

## Abstract

**Purpose:**

Patients with acute coronary syndrome (ACS) face various challenges in the physical, psychological, social and functional domains of their lives, with adverse effects on their quality of life. The present study evaluated the effects of a smartphone-based self-help intervention on the quality of life of patients with ACS.

**Design:**

A randomized controlled trial.

**Methods:**

Sixty-four ACS patients were selected via convenience sampling and randomly assigned to one of the intervention or control groups (32 participants in each group). The participants in the intervention group received a smartphone**-**based self-help intervention in six sessions for three weeks. The smartphone-based self-help intervention involved six educational sessions delivered via WhatsApp, covering disease-related knowledge, emotional regulation, cognitive restructuring, sleep hygiene, resilience, and self-care practices. Patients accessed the material asynchronously and applied it to their daily routines. Data were collected before, immediately after, and three weeks after the intervention via a demographic questionnaire and the Ferrans and Powers Quality of Life Index (QLI). The collected data were analyzed in SPSS, version 22.

**Results:**

There were no statistically significant differences between the two study groups regarding the quality-of-life mean scores measured before (*p* = 0.92) and immediately after (*p* = 0.26) the intervention. Three weeks after the intervention, however, the intervention group’s mean quality of life score was greater than that of the control group (*p* = 0.03). The results of the repeated-measures ANOVA revealed that the mean quality-of-life scores in the intervention group increased, whereas the mean quality-of-life scores in the control group remained stable.

**Conclusion:**

The results of this study indicated that a smartphone-based self-help intervention effectively improved the quality of life of patients with ACS. Nurse managers and clinical nurses can utilize these findings to increase the quality of life of patients.

**Trial registration:**

This trial was prospectively registered in the Iranian Registry of Clinical Trial (IRCT20220424054627N1) on 15 May 2022.

**Supplementary Information:**

The online version contains supplementary material available at 10.1186/s12872-025-05060-5.

## Introduction

Cardiovascular diseases (CVDs) are among the most serious chronic diseases because CVDs are the leading causes of morbidity and mortality worldwide [1]. It is estimated that one out of three Americans will be affected by cardiovascular disease by 2030. In Iran, 6.4 out of 10,000 deaths are due to cardiovascular diseases [2, 3]."Acute coronary syndrome (ACS) is one of the most common and dangerous manifestations of CVDs, encompassing a spectrum of ischemic cardiac conditions, from unstable angina to myocardial infarction.", with an estimated 23 million deaths worldwide expected by 2030 [4, 5]. ACS remains the leading cause of morbidity and mortality in Iran, with over 600,000 deaths attributed to it annually [6].

Patients with ACS are at high risk for serious complications, such as arrhythmia and hemodynamic instability. Invasive procedures, including percutaneous coronary intervention and coronary artery bypass grafting, may be necessary for the treatment of ACS. However, these procedures can cause significant stress and anxiety in patients [7]. Patients with ACS often experience a decreased health-related quality of life, which may be due to disease severity and psychological stress [8]. Quality of life (QOL) is defined by the World Health Organization as"an individual's perception of their position in life in the context of the culture and value systems in which they live and in relation to their goals, expectations, standards and concerns"[9]. The literature shows that if patients'quality of life decreases following an ACS diagnosis, they rarely return to their predisease level of performance [10]. The results of a study conducted in Saudi Arabia indicated that ACS patients experienced significant impairments in all areas of quality of life, including emotional, physical, and social dimensions [11]. Currently, considering the increasing prevalence of ACS and its impact on the physical, psychological and social aspects of patients’ lives, ACS patients’ quality of life has become very important. Quality of life is also an important criterion for evaluating the effects of treatments [12]. In the context of acute coronary syndrome, treatments such as cardiac rehabilitation programs, psychological counseling, and patient education interventions have been evaluated based on their impact on quality-of-life outcomes [13]. These approaches aim not only to reduce recurrence or mortality but also to improve physical functioning, emotional well-being, and social participation.

There are various methods used to increase the quality of life of these patients. These interventions focus mainly on the acceptance of disease, treatments, and self-care [14]. Self-help is one of these interventions and is designed to increase patients’ access to health-improving interventions [15].

Self-help is a standardized type of psychological therapy in which patients can apply independently anytime and anywhere, Thus, the educational content will be constantly available to patients, who can easily apply it in their self-care routines [16]. In self-help interventions, there is no face-to-face interaction between patients and healthcare professionals, and if there is a need for any communication between the two, it will be only supportive and educational in nature [17]. Most self-help interventions are designed on the basis of the principles of cognitive behavioral therapy (CBT) [18]. The purpose of CBT is to change patients’ behaviors through modifying their thinking patterns and eliminating negative emotions. For patients with ACS, CBT can increase patients’ knowledge, improve health-related behaviors, and improve disease management [19]. However, several factors have limited many patients'accesses to CBT. These include a shortage of qualified healthcare professionals, high costs, fear of stigma, long wait times to see a psychologist, and the need to travel long distances for some patients. One potential solution to this issue is the implementation of self-help interventions on the basis of the principles of CBT [20, 21]. Self-help interventions can be implemented via books and booklets; CDs and DVDs; and multimedia, internet and smartphone apps [22]. In recent years, a great deal of attention has been given to self-help interventions that use internet and smartphone apps [23]. Smartphone-based self-help interventions provide inexpensive, useful, and evidence-based care to a wide variety of patients and reduce healthcare costs [24]. In a smartphone-based self-help intervention, structured educational content that has been developed on the basis of the principles of CBT is divided into weekly lessons and then presented to patients via internet and smartphone apps [25]. Thus, the educational content will be constantly available to patients, who can easily apply it in their self-care routines [16].

The effectiveness of self-help interventions has been evaluated for various medical conditions, including tinnitus [26], cardiovascular disease [27, 28], insomnia [29], chronic pains [30], digestive disorders [31], and obesity [32], but researchers have not identified studies that have evaluated the effects of smartphone-based self-help interventions in ACS patients.

Previous nursing research has demonstrated the potential of smartphone-based interventions in managing chronic conditions such as heart failure, diabetes, and hypertension. These interventions, often developed or implemented by nurses, have been associated with improved adherence, reduced anxiety, and better quality of life. For example, Davoudi et al. (2020) found significant improvements in QOL in heart failure patients following a nurse-led smartphone intervention [33]. Matias et al. (2021) stated that in patients with acute coronary syndromes, a smartphone-based cardiac rehabilitation program, as a supplement to usual care, improved exercise capacity in 8 weeks, in addition to participation and adherence to cardiac rehabilitation [34]. Other studies also provide evidence on the feasibility and usefulness of innovative smartphone-phone based platforms in addressing treatment and prevention gaps in patients with acute coronary syndromes [35, 36]. Such evidence supports the integration of digital strategies in nursing protocols for ACS care.

Therefore, considering the importance of improving the quality of life of patients with ACS, the present study was conducted to evaluate the effect of a smartphone-based self-help intervention on the quality of life of patients with ACS.

## Methods

### Patient and public involvement

#### Trial design

Trial registration: This study was a prospectively registered randomized controlled trial. The protocol was approved by the ethics committee of Shiraz University of Medical Sciences (ethics code: IR.SUMS.NUMIMG.REC.1401.008) and registered in the Iranian Registry of Clinical Trials (IRCT) with ID IRCT20220424054627N1 on 15 May 2022 (accessible at: https://irct.behdasht.gov.ir/search/result?query=IRCT20220424054627N1).

This is a randomized controlled trial conducted on patients with ACS in Gerash County in southern Fars Province, Iran. The participants were selected through convenience sampling and randomly assigned to one of the intervention or control groups (32 participants in each group). The random sequence was generated using the Random Allocation software, and the statistical consultant, who is one of the authors, was responsible for its creation and implementation. Additionally, all blocks were fully completed.

##### Trial setting

This study conducted on patients with ACS in Gerash County in southern Fars Province, Iran.

##### Eligibility criteria for participants

Participants must have a confirmed diagnosis of acute coronary syndrome (ACS) by a cardiologist, be at least 24 h into their hospital stay with this diagnosis, and be between 30 and 65 years of age. Additionally, participants needed to be willing to participate in the study, be able to read and write in Farsi, have the ability to use smartphones, and have access to the internet. Those who had participated in psychological interventions in the past three months were also excluded. Furthermore, patients who chose to withdraw from the study, required coronary artery bypass graft surgery for ACS, developed life-threatening complications such as arrhythmia, or passed away during the study were not included.

##### Intervention and comparator

After the participants were divided into an intervention group and a control group, the researchers introduced a smartphone-based self-help intervention consisting of six sessions over three weeks. The educational content, organized into six main topics, was compiled into a booklet and sent to the participants in the intervention group via WhatsApp twice a week (two topics per week).

The WhatsApp platform was used because it was available and easy to use. Furthermore, due to the constraints imposed by the COVID-19 outbreak, which rendered in-person access to patients unfeasible, the researchers opted to disseminate educational materials to patients via an online platform (WhatsApp). Our analysis showed that 100% of participants actively accessed the materials at least two times per week.

##### Outcomes

The quality of life of the participants in both groups was measured via the QLI before, immediately after, and three weeks after the intervention [37]. To evaluate the effectiveness of the intervention, the quality-of-life mean scores obtained at each stage of the study were compared between the intervention and control groups. In line with ethical principles of research, the educational booklet was provided to the participants in the control group after the completion of the study.

##### Sample size

The sample size was calculated via the following formula and in a study by Schneider et al. [25]; 32 patients were included in each group.

$$\mathbf{n}={\varvec{\uprho}}\frac{{({\mathbf{z}}_{1-\frac{{\varvec{\upalpha}}}{2}}+{\mathbf{z}}_{1-{\varvec{\upbeta}}})}^{2}{({\mathbf{S}}_{1}+{\mathbf{S}}_{2})}^{2}}{{({{\varvec{\upmu}}}_{1}-{{\varvec{\upmu}}}_{2})}^{2}}$$

α = 5%

1-β = 80%

µ_1_ = 34.13

µ_2_ = 25.58

S_1_ = 20.48

S_2_ = 13.24

N = 32

##### Randomization

Initially, the researcher contacted Amiralmomenin Hospital in Gerash after the ethics code was obtained. A nurse provided information about the study to potential participants, and 64 eligible patients who met the inclusion criteria were subsequently enrolled. A block random allocation method was employed to assign patients to the control and intervention groups. To create this sequence, the total sample size of 64 patients was divided into 16 blocks, with 4 patients in each block. On the basis of the generated random sequence, two participants in each block were assigned to the intervention group, whereas the other two were assigned to the control group. The objectives and methods of the study were then explained to the patients. Those willing to participate were asked to complete an informed consent form. In the next stage, the participants completed a demographic questionnaire and a quality-of-life questionnaire for pretest assessment. The fourth researcher who performed the data analysis was blinded to group assignment.

### Outcome measures

A demographic information questionnaire was prepared based on a review of the literature and the opinion of the research team, which contained items related to age, sex, marital status, occupation, medical insurance, smoking, and drug use.

This questionnaire addresses quality of life in the physical, socioeconomic, and family domains. The QLI consists of two parts, each containing 35 items. The first part measures the level of importance that patients assign to different aspects of their lives, whereas the second part evaluates their satisfaction with these aspects. The scoring is based on a 6-point Likert scale ranging from 1 to 6. In the importance section, higher scores indicate greater importance; similarly, in the satisfaction section, higher scores indicate greater satisfaction. The scoring procedure for the QLI is as follows. For calculating final score, first, satisfaction scores are shifted to center around zero by subtracting 3.5. Then, each satisfaction score is multiplied by its corresponding importance score. These weighted values are added together to get a total score, which is divided by the number of responded items. The final score ranges from −15 to + 15, and any negative scores are eliminated by adding 15 to the total score. The score range on the QLI is between 0 and 30, with higher scores representing better quality of life [38]. Ferrans and Powers (1992) reported a Cronbach's alpha of 0.93 for the English version of this questionnaire [39]. Cronbach's alpha for the Persian version of the QLI was evaluated by Shojaei et al. [40] and reported to be 0.86.

**Fig. 1:**
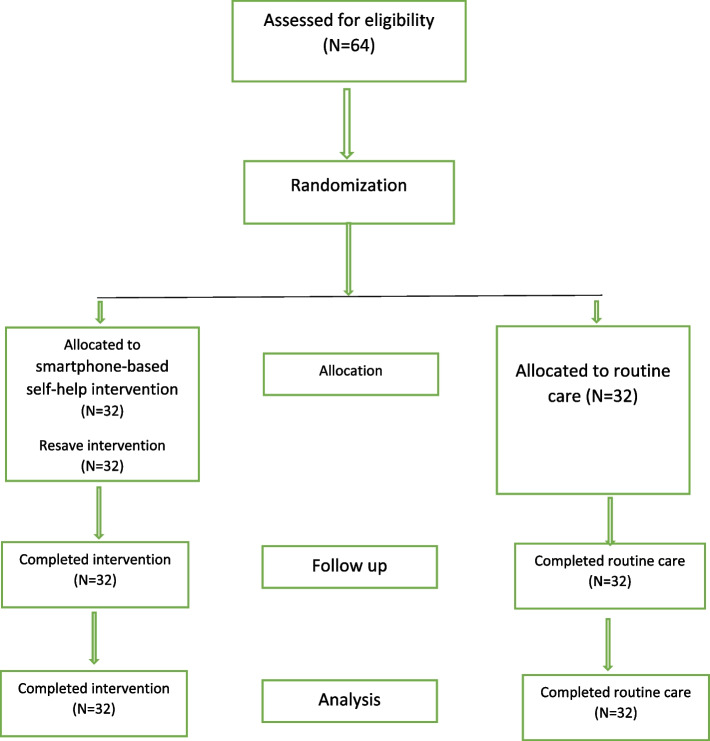
Consort flow chart of the study

Manuscript was prepared in accordance with the CONSORT reporting guidelines for randomized controlled trials and the procedural steps for conducting the study are illustrated in Fig. 1.

The topic of interventions is listed in Table 1.

**Table 1:** Educational content of each session

Topic	Educational content
1. The disease	Information about acute coronary syndrome and its effects on health and quality of life, including suitable physical activities for patients with ACS, dietary recommendations, drug regimens, and guidance on how often patients should visit their doctors
2. Understanding one’s emotions and feelings	facing and coping with negative emotions related to ACS, such as Information on anxiety, helplessness, anger, guilt and depression and strategies to deal with these feelings
3. Attitude change	reconstruction of thought patterns and cognitive thinking, such as Information about negative attitudes, unpleasant thoughts, rumination, irrational beliefs, and methods of reconstruction of thought patterns
4. Improving resilience	methods for improving resilience and coping strategies, such as Information about the importance of resilience and three methods for improving resilience, namely, effective coping, enjoying life, and positive thinking
5. Sleep and rest	introduced the participants to sleep hygiene and relaxation techniques, such as Information about sleep, sleep hygiene, deep breathing technique, and progressive muscle relaxation
6. Self-care	emphasized the importance of self-care and personal development, such as Information about personal development, importance of enjoying life, importance of being grateful, and mindfulness

The participants were encouraged to study the educational content and apply it to their daily care. They could also contact the researcher via phone or text messaging to ask any questions they had about the education and how to implement it in their self-care. In contrast, the patients in the control group received only routine care, which included examinations by doctors and education from healthcare professionals.

The collected data were analyzed via SPSS version 22, Data normality was assessed using the Shapiro–Wilk test and visual inspection of Q-Q plots. The results indicated that the assumption of normality was met for the quality-of-life scores, justifying the use of parametric tests, including independent t-tests and repeated measures ANOVA. Additionally, degrees of freedom and sample sizes for each statistical test have now been explicitly reported to ensure transparency in the analysis.

### Statistical analysis

Therefore, independent sample t tests and chi-square tests were used for demographic data comparisons, and repeated-measures ANOVA was used to evaluate quality-of-life scores between two groups over time.

### Ethical considerations

The present article is part of a thesis for a master’s degree in nursing and has been approved by the ethics committee of Shiraz University of Medical Sciences (ethics code: IR.SUMS.NUMIMG.REC.1401.008) and the Iranian Registry of Clinical Trials (IRCT code: IRCT20220424054627N1. 2022.05.15), available at https://irct.behdasht.gov.ir/search/result?query=IRCT20220424054627N1.

### Trial registration

This trial was prospectively registered in the Iranian Registry of Clinical Trial (IRCT20220424054627N1) on 15 May 2022.

## Results

A total of 64 patients with ACS participated in this study (32 participants in the control group and 32 participants in the intervention group). The mean age of the participants was 55.38 ± 8.39 years, and 39.1% of the participants were male. The results revealed that there were no statistically significant differences between the intervention and control groups in terms of demographic data. The groups’ demographic characteristics are shown in Table 2.

**Table 2:** Comparison of the intervention and control groups’ demographic characteristics

Variable		Intervention group	Control group	Total N (%)	*p* value
		**(Mean ± SD)**	**(Mean ± SD)**		
Age***		53.84 ± 8.58	56.91 ± 8.04		0.146
		N (%)	N (%)		
Gender^**^	Male	13 (40.6)	12 (37.5)	25 (39.1)	0.798
	Female	19 (59.4)	20 (62.5)	39 (60.9)	
	Total	32 (100)	32 (100)	64 (100)	
Marital status^*^	Single	3 (9.4)	1 (3.1)	4 (6.2)	0.634
	Married	22 (68.8)	23 (71.9)	45 (70.3)	
	divorced	1 (3.1)	0 (0)	1 (1.6)	
	Widowed	6 (18.8)	8 (25)	14 (21.9)	
	Total	32 (100)	32 (100)	64 (100)	
Education level^*^	Under high school	20 (62.5)	26 (81.2)	46 (71.9)	0.43
	High-school Diploma	7 (21.9)	5 (15.6)	12 (18.8)	
	Bachelor’s degree	2 (6.2)	0 (0)	2 (3.1)	
	Master's degree	2 (6.2)	1 (3.1)	3 (4.7)	
	P.H.D	1 (3.1)	0 (0)	1 (1.6)	
	Total	32 (100)	32 (100)	64 (100)	
Occupation^*^	Unemployed	2 (6.2)	4 (12.5)	6 (9.4)	0.401
	Self-employed	9 (28.1)	8 (25)	17 (26.6)	
	Housewife	14 (43.8)	18 (56.2)	32 (50)	
	Retired	5 (15.6)	2 (6.2)	7 (10.9)	
	Employee	2 (6.2)	0 (0)	2 (3.1)	
	Total	32 (100)	32 (100)	64 (100)	
Underlying disease^**^	Yes	12 (37.5)	12 (37.5)	24 (37.5)	1.00
	No	20 (62.5)	20 (62.5)	40 (62.5)	
	Total	32 (100)	32 (100)	64 (100)	
Type of underlying disease^*^	No underlying disease	20 (62.5)	20 (62.5)	40 (62.5)	0.427
	Diabetes	6 (18.8)	8 (25)	14 (21.9)	
	Hypertension	4 (12.5)	1 (3.1)	5 (7.8)	
	Respiratory diseases	0 (0)	2 (6.2)	2 (3.1)	
	Renal diseases	2 (6.2)	1 (3.1)	3 (4.7)	
	Total	32 (100)	32 (100)	64 (100)	
Drug addiction^*^	Yes	2 (6.2)	3 (9.4)	5 (7.8)	1.00
	No	30 (93.8)	29 (90.6)	59 (92.2)	
	Total	32 (100)	32 (100)	64 (100)	
Smoking (cigarettes and water-pipe)^**^	Yes	11 (34.4)	12 (37.5)	23 (35.9)	0.794
	No	21 (65.6)	20 (62.5)	41 (64.1)	
	Total	32 (100)	32 (100)	64 (100)	

^*^Fisher’s exact test

^**^Chi-square test

^***^independent sample t test

The independent samples t test results revealed that there was no statistically significant difference between the intervention and control groups’ mean quality of life scores before (*p* = 0.92) and immediately after the intervention (*p* = 0.26).

Three weeks after the intervention, however, the intervention group’s mean quality of life score was significantly greater than that of the control group (*p* = 0.03). Additionally, the mean values of the changes in the intervention and control groups’ quality of life scores both immediately and three weeks after the intervention were significantly different (*p* < 0.001).

Although immediate post-intervention comparisons revealed no statistically significant intergroup differences in absolute quality-of-life scores (*p* = 0.26), the marked improvement from baseline measurements (*p* < 0.001) implies a potential latency or cumulative therapeutic effect. This phenomenon became more pronounced at the three-week follow-up assessment, where both absolute scores (*p* = 0.03) and longitudinal changes maintained statistical significance.

These results are shown in Table 3.

**Table 3:** Comparisons between the two groups’ mean quality-of-life scores and mean values of changes in quality-of-life scores

Variable	Stage	Intervention group (mean ± SD)	Control group (mean ± SD)	Independent samples T test results	
				**T**	***p* value**
Quality of life	Before intervention	16.81 ± 6.37	16.65 ± 6.89	−0.094	0.92
	Immediately after intervention	18.25 ± 6.09	16.45 ± 6.63	−1.12	0.26
	3 weeks after intervention	19.76 ± 5.84	16.45 ± 6.33	−2.17	0.03
Mean value of changes in quality of life	Immediately after intervention	1.43 ± 1.51	−0.20 ± 0.86	−5.33	< 0.001
	3 weeks after intervention	2.95 ± 1.71	−0.20 ± 1.09	−8.78	< 0.001

The results of the repeated measures analysis of variance test indicated that the quality-of-life score in the experimental group showed a significant trend over time, whereas the quality-of-life score in the control group did not change significantly over the same period. The details are given in Table 4.

**Table 4:** Comparisons of the mean quality of life scores between the two control and intervention groups over time

Source of changes	Sum of squares	df	Mean of squares	F	Effect size	*P*_Value
Time	0.880	1.65	0.44	1.05	0.01	0.35
Group	139.56	1.56	69.74	66.97	0.10	< 0.001
Error	25.95	51.23	0.50	-	-	-
Within subject contract (Intervention group)	139.53	1	139.53	95.40	0.62	< 0.001
Within subject contract (control group)	0.66	1	0.66	1.09	0.23	0.30

Pairwise comparisons of quality-of-life scores between groups at different time points (Bonferroni-corrected) appear in Table 5.

**Table 5:** Pairwise comparisons of the mean quality of life score at the time points studied, by intervention and control groups, via the Bonferroni correction

Group	Time*	Mean difference	95% CI		*p* value
			Lower	Upper	
Intervention	Time 1, 2	−1.43	−2.11	−0.76	< 0.001
	Time 1, 3	−2.95	−3.71	−2.18	< 0.001
	Time 2, 3	−1.51	−1.97	−1.05	< 0.001
Control	Time 1, 2	0.15	−0.18	0.58	0.57
	Time 1, 3	0.19	−0.28	0.69	0.91
	Time 2, 3	0.13	−0.33	0.33	1

^*^Time period 1: before intervention, 2: immediately after intervention, 3: three weeks after intervention

The quality-of-life score comparison pair table displays a calculation of each participant’s quality-of-life score: subtracting the second measurement (post-intervention) from the first measurement (pre-intervention).

The results of the table above show that the average quality of life scores in the intervention group was significantly different from each other at all three time points: before the intervention, immediately after the intervention, and three weeks after the intervention. The average quality of life scores in the control group were not significantly different at any of the three time points: before the intervention, immediately after the intervention, or three weeks after the intervention.

### Harm

No risks or threats were observed or reported by participants in this study.

## Discussion

The results of the present study showed that the smartphone-based self-help intervention was effective in improving the quality of life of patients with ACS. The self-help intervention was designed on the basis of the principles of cognitive behavioral therapy.

The absence of immediate importance in overall scores suggests that the impact of the intervention takes time to reveal itself. This is consistent with cognitive and behavioral approaches that typically require prolonged participation and effort before any tangible benefits are evident. The cumulative effect observed is in line with the hypothesis that patients’s quality of life can be incrementally improved, over time, through smartphone-based self-help interventions rather than through drastic changes immediately following the intervention.

In a similar study by Schneider et al. [41], a self-help intervention was found to improve the quality of life of patients with irritable bowel syndrome six months after the intervention. In a clinical trial conducted by Hunt et al. in 2020 [42], the effects of a self-help intervention on the quality of life of patients with inflammatory bowel disease were investigated. The self-help intervention was designed on the basis of the principles of CBT and in the form of an educational booklet. The results of this study confirmed the role of self-help interventions in enhancing patients'quality of life, which aligns with the findings of the present study. The results of a meta-analysis in 2024 revealed that self-help interventions may have positive effects on diabetes distress, anxiety, self-management behavior, and quality of life [43]. The results of another study in Iran confirmed that the use of a smartphone-based app versus routine care can improve the quality of life of patients with heart failure [33]. The results from another Australian study showed that in patients with ACS, a smartphone-based cardiac rehabilitation program, as an adjunct to UC, improved exercise capacity at 8 weeks in addition to participation in and adherence to cardiac rehabilitation [34]. Currently, the use of modern educational methods, including smartphones, is a very suitable method for education and information transfer. Additionally, among the problems that patients face is confusion about the disease and the continuation of life and the concerns related to it. Therefore, providing education to patients using smartphones, which is a convenient and accessible method in terms of time and cost for patients, can be an acceptable method for educating patients and increasing their quality of life. In addition, patients have the opportunity to study and use educational materials at a time that is convenient for them.

In contrast to the results of our study, the results of Wang et al.’s (2018) study showed that self-help intervention had little effect on the quality of life of patients immediately and three months after the intervention [19]. This discrepancy could be due to differences between the methodologies of the studies (an online intervention against face-to-face education), the durations of the interventions (3 weeks versus 4 weeks) and the posttest measurements (3 weeks versus 3 months). Furthermore, the most important difference could be related to the populations studied.

From a nursing perspective, this study supports the feasibility and effectiveness of remotely delivered, structured educational interventions in improving patients'quality of life. The findings advocate for integrating digital health tools into routine nursing practice, particularly for follow-up education and self-care support among patients with cardiovascular conditions. Nurses can play a crucial role in guiding patients in using digital interventions effectively and adapting content to individual needs.

## Conclusion

The results indicated that the smartphone-based self-help intervention significantly enhanced the quality of life for patients with acute coronary syndrome (ACS).

From a nursing perspective, from a nursing perspective, increasing patients'knowledge and awareness about their disease can increase their quality of life. also, this study supports the feasibility and effectiveness of remotely delivered, structured educational interventions in improving patients'quality of life. The findings advocate for integrating digital health tools into routine nursing practice, particularly for follow-up education and self-care support among patients with cardiovascular conditions. Nurses can play a crucial role in guiding patients in using digital interventions effectively and adapting content to individual needs.

Other studies have stated that A nurse-led smartphone-based self-management program is as effective as direct hospital-provided patient care in improving outcomes for most. A nurse-led smartphone-based self-management program can free up valuable time for nurses to provide care to critically ill patients [9, 44, 45].

Consequently, the researchers advocate for further studies to investigate the effects of self-help interventions on the quality of life of patients with cardiac disorders and other chronic diseases. Additionally, they recommend comparative analyses between online self-help interventions and face-to-face educational interventions and other educational methods and other disease.

## Strengths and limitations

In the present study, self-help interventions were implemented via the internet and smartphones. This approach minimized unnecessary contact with participants during the COVID-19 pandemic and offered several advantages, including time savings, reduced costs, and convenience for both the research team and the patients. With this self-help intervention, patients can study educational content anytime and anywhere, easily applying the instructions to their routine self-care activities. Another strength of this study is that the self-help intervention was developed according to the principles of cognitive behavioral therapy.

One restriction for this study is the lack of complete blinding for participants as well as all researchers. We accept that full blinding was not feasible for the participants and the staff administering the intervention. To reduce bias, a group allocation blind was enforced at the outcome evaluation level. Furthermore, although random allocation was done for sample distribution, the initial sampling method was random because of restricted access to patients. More specifically, the chosen convenience sampling technique does not reflect the broader population of patients with ACS. Furthermore, another potential limitation is assuming some eligible patients who do not have smartphones were excluded. The aforementioned limitations, particularly convenience sampling and exclusion of patients without smartphones, may reduce the generalizability of the findings to disadvantaged populations or those with limited access to technology. For example, patients on low incomes or in rural areas may be less likely to have access to smartphones, and therefore the present intervention may not be applicable to them. Also, the lack of complete blinding could affect the internal validity of the study, and therefore, generalization of the results should be made with caution. To enhance generalizability, future studies could include participants from underserved populations by utilizing simplified technologies such as SMS-based interventions or printed educational materials, allowing participation even among those without smartphones or internet access.

Also, the focus on change scores rather than absolute post-intervention scores illustrates the need to apply more focus on a behavioral intervention’s cumulative effects. These findings may need confirmation in future studies that use larger sample sizes and extended follow-up durations to test changes in those diagnosed with ACS. Lastly, underscoring these study limitations is a relatively small sample size that, while useful for indicating change, may explain the low baseline inter-group similarity (*p* = 0.92). Finally, the reliance on change scores rather than absolute post-intervention scores highlights the importance of considering cumulative effects in behavioral interventions. Future studies with larger sample sizes and longer follow-up periods are recommended to further validate these findings.

## Supplementary Information

Supplementary Material 1

## Data Availability

Data is provided within the manuscript.

## Abbreviations

ACS: Acute Coronary Syndrome
CBT: Cognitive Behavioral Therapy
CVD: Cardiovascular Disease
IRCT: Iranian Registry of Clinical Trials
QoL: Quality of Life
QLI: Quality of Life Index
RCT: Randomized Controlled Trial
SD: Standard Deviation
SPSS: Statistical Package for the Social Sciences
